# *PPP1R21*-Related neurodevelopmental disorder: Phenotypic delineation, variant spectrum, and pathophysiological mechanisms

**DOI:** 10.1007/s10048-026-00920-4

**Published:** 2026-08-08

**Authors:** Francesco Fabrizio Comisi, Gabriele Di Pasquale, Andrea Maria Comisi, Giuseppe Donato Mangano, Piero Pavone, Alessandro Ferretti, Vincenzo Salpietro, Pasquale Parisi, Alberto Spalice

**Affiliations:** 1 Pediatric Clinic and Rare Diseases, Microcitemico Hospital “A. Cao”, University of Cagliari, 09124 Cagliari, Italy; 2 Department of Biotechnological and Applied Clinical Sciences, Academic Unit of Pediatrics, University of L’Aquila, L’Aquila, Italy; 3 Department of Clinical and Experimental Medicine, University of Catania, 95131 Catania, Italy; 4 Department of Medicine and Surgery, Kore University of Enna, Enna, Italy; 5 Unit of Pediatrics, and Psychiatric Department of Child and Experimental Medicine, University of Catania, A.O.U. Policlinico G. Rodolico, Catania, Italy; 6 Department of Neuroscience, Mental Health and Sensory Organs (NESMOS), Sant’Andrea University Hospital, Sapienza University of Rome, Rome, Italy; 7 European Brain Research Institute “Rita Levi-Montalcini”, Viale Regina Elena, Rome, Italy; 8 Department of Maternal, Infantile and Urological Sciences, La Sapienza University of Rome, 00161 Rome, Italy

**Keywords:** *PPP1R21*, Neurodevelopmental disorder, FERRY complex, Endosomal dysfunction, Intellectual disability, Hypomyelination

## Abstract

*PPP1R21*-related neurodevelopmental disorder (*PPP1R21*-NDD) is an ultra-rare autosomal-recessive encephalopathy caused by dysfunction of the Five-subunit Endosomal Rab5 and RNA/ribosome intermediarY (FERRY) complex. To delineate the clinical, neuroimaging, and molecular spectrum of PPP1R21-NDD and outline diagnostic and research priorities. Targeted searches of PubMed, EMBASE, Scopus, Web of Science, and Google Scholar through January 2026 were performed. Eligible reports included molecularly confirmed biallelic PPP1R21 cases and related functional studies. Two researchers independently extracted individual-level data following narrative review quality criteria. Twenty-five individuals from 21 families harbored 17 distinct variants (15 loss-of-function, 2 missense), all homozygous, reflecting high consanguinity. Profound global developmental delay/intellectual disability was universal; hypotonia near-universal (22/25, 88%). Ambulation was delayed and ataxic when achieved; expressive language was minimal or absent. A recognizable coarse facial gestalt was observed with thick eyebrows, broad nasal bridge, thick lips, and low-set ears. Systemic features included feeding dysfunction and respiratory morbidity requiring gastrostomy or tracheostomy in severe cases. Mortality was 17% (4/24). Neuroimaging showed callosal thinning, white-matter volume loss, ventricular enlargement, and vermian hypoplasia. Patient fibroblasts showed delayed transferrin clearance and elevated proteasome activity, suggesting endosomal dysfunction and proteasome hyperactivation. PPP1R21-NDD is a severe recessive encephalopathy with a convergent phenotype and evidence of endo-lysosomal dysfunction. Diagnosis should be pursued with exome or genome sequencing in patients with characteristic clinical and neuroimaging features, particularly in consanguineous families. Research priorities include natural history studies, standardized magnetic resonance imaging (MRI) protocols, neural disease models, and therapeutic strategies targeting endosomal trafficking.

**Supplementary Information:**

The online version contains supplementary material available at 10.1007/s10048-026-00920-4.

## Introduction

### Background & clinical rationale

*PPP1R21*-related neurodevelopmental disorder (Protein Phosphatase 1 Regulatory subunit 21; Online Mendelian Inheritance in Man [OMIM] #619383) is an autosomal-recessive encephalopathy marked by severe intellectual disability (ID), hypotonia, a coarse craniofacial gestalt, and structural brain abnormalities [1–6]. *PPP1R21* variants were first implicated in ID within a large cohort of individuals with cognitive impairment described in 2017, and published cases remain few (≈ two dozen by 2025) [1–3, 5–10]. The disorder illustrates the diagnostic yield of next-generation sequencing, particularly whole-exome sequencing, in consanguineous populations [2, 5, 7, 11–13]. Beyond rarity, its significance lies in mechanistic insight: converging evidence implicates early endosomal trafficking and putative endosome-coupled local messenger RNA (mRNA) translation in neurons [1, 2, 4, 14, 15]. Accordingly, *PPP1R21*, *TBCK*, and *C12orf4* have been proposed as a shared “FERRY complex” disease class with overlapping neurodevelopmental features. Comparative delineation of those disease entities remains limited and recent reviews have outlined open questions regarding this putative disease spectrum [4, 14, 15]. Key gaps include incomplete adult phenotyping, limited genotype–phenotype correlation, uncertain links between FERRY dysfunction and clinical manifestations, and the lack of targeted therapeutic approaches.

### *PPP1R21* and the FERRY complex

PPP1R21 protein forms the structural backbone of the five-subunit FERRY complex and binds Rab5-GTP on early endosomes. The complex is proposed to couple early endosomal transport with the shuttling of specific mRNAs to enable local translation, particularly in neurons. This positioning provides a mechanistic link between endosomal trafficking and neurodevelopmental phenotypes in *PPP1R21* deficiency [4, 14–17].

### Objectives

This review integrates the clinical, neuroimaging, and molecular spectrum of *PPP1R21*-related neurodevelopmental disorders with current understanding of its underlying molecular mechanisms, providing pragmatic diagnostic and management guidance and outlining priorities for future translational studies.

## Methods

This narrative review synthesizes the literature on *PPP1R21*-associated neurodevelopmental disorders. PubMed, EMBASE, Scopus, Web of Science, and Google Scholar were searched through January 2026, combining *PPP1R21* (and its full designation, protein phosphatase 1 regulatory subunit 21) and “FERRY complex” with terms for neurodevelopmental disorder, intellectual disability, and hypotonia; the reference lists of relevant articles were hand-searched. Eligible records were original reports of molecularly confirmed biallelic *PPP1R21* cases, functional studies, and pertinent reviews; heterozygous-only descriptions and duplicate cases were excluded; the included individuals and the publication-by-family map used to reconcile overlapping reports are provided in Online Resources 1 and 2, respectively. Two researchers (F.F.C., G.D.P.) independently screened, extracted, and reconciled the data using a standardized template, with variants annotated in Human Genome Variation Society (HGVS) nomenclature on the Matched Annotation from NCBI and EMBL-EBI (MANE) Select transcript NM_001135629.3 (GRCh38). Because this evidence base comprises almost exclusively case reports and small series, a narrative synthesis was adopted. The review was structured to meet the Scale for the Assessment of Narrative Review Articles (SANRA) quality criteria [18], qualitatively integrating clinical, molecular, and functional evidence. No large language models or artificial intelligence-based tools were used for content generation in this manuscript.

## *PPP1R21*

### Locus and protein architecture

*PPP1R21* maps to 2p16.3 (GRCh38: chr2:48,440,766–48,515,386), spans ~ 74.6 kb [1, 10]. Three RefSeq transcripts (the MANE Select NM_001135629.3, encoding NP_001129101.1, plus NM_152994.5 and NM_001193475.2) comprise 22, 21, and 20 exons, respectively; all three are identical through exon 15 and diverge at the 3′ end due to in-frame alternative splicing. The longest isoform encodes a ~ 780-aa coiled-coil protein that provides the structural scaffold of the FERRY complex [1, 4, 14, 15]. Structural analyses depict an elongated assembly with PPP1R21 coiled-coils projecting from a clamp-like core [4, 15]. Two conserved regions are recognizable, an N-terminal KLRAQ segment (aa 11–111) and a C-terminal TTKRSYEDQ segment (aa 255–771), both preserved from nematodes to humans [1, 4].

### Tissue and central nervous system expression

*PPP1R21* transcripts are detectable in multiple organs (brain, endocrine tissues, lung, liver, heart, pancreas, spleen, gastrointestinal tract), with highest protein abundance reported in cerebellum, esophagus, pancreas, thyroid, and parathyroid. Embryonic mouse cortex (E16) immunofluorescence supports a role of this gene in neurodevelopmental processes [1]. Human Protein Atlas data indicate moderate neuronal expression across the central nervous system with enrichment in Purkinje cells and deep cerebellar nuclei, and single-cell RNA-seq shows expression in neuronal and glial lineages across early brain development [12, 19].

### Role in cellular function

**Fig. 1:**
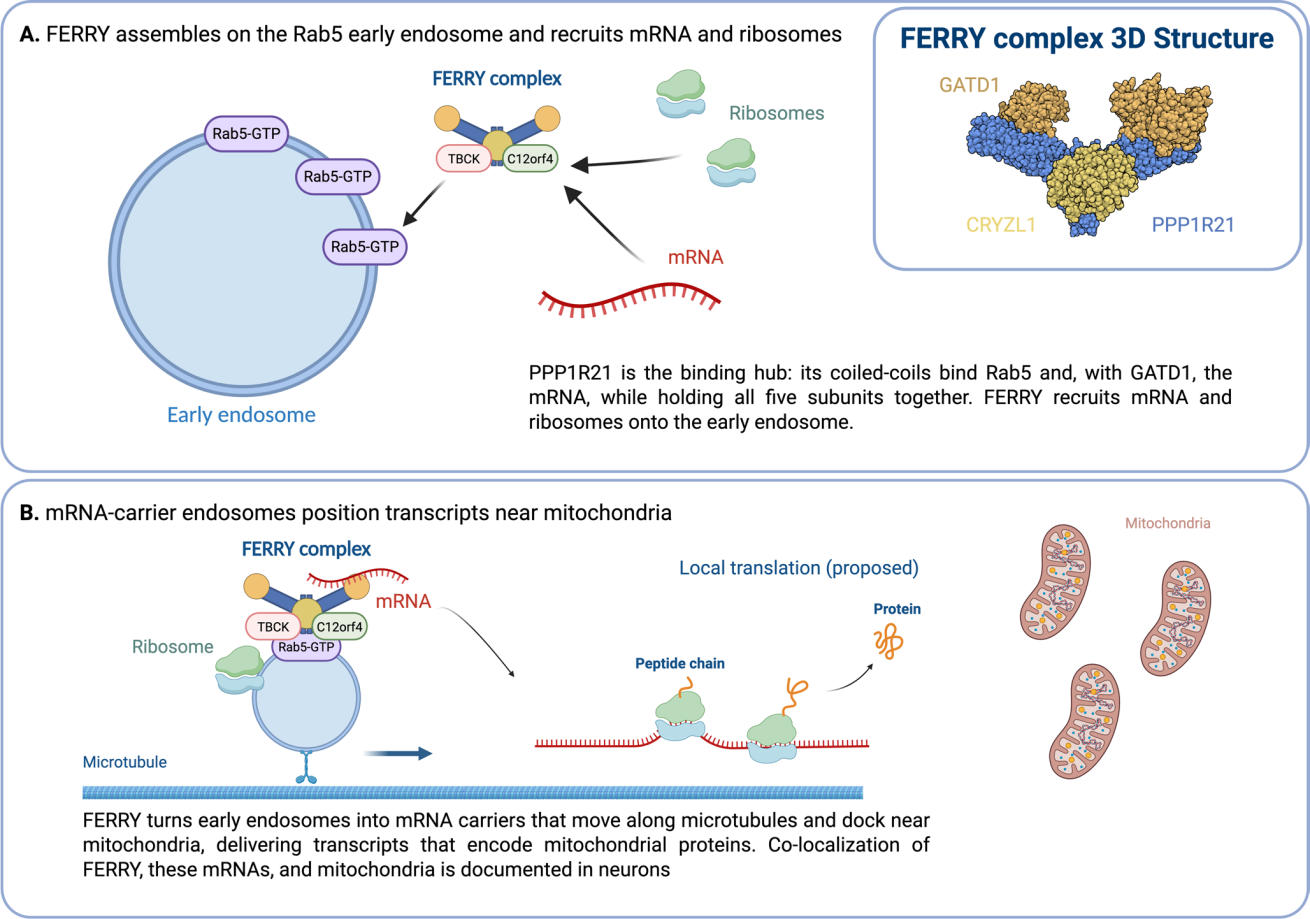
**Proposed structure and function of the FERRY complex**. (**A**) FERRY assembles on the Rab5-positive early endosome: PPP1R21, the structural backbone, binds Rab5-GTP and, together with GATD1, the mRNA, while holding all five subunits (PPP1R21, TBCK, C12orf4, CRYZL1, GATD1) together; the complex recruits both mRNA and ribosomes onto the endosome. (**B**) mRNA-carrier early endosomes move along microtubules and dock near mitochondria, delivering transcripts that encode mitochondrial proteins (e.g., MDH2); co-localization of FERRY, these mRNAs, and mitochondria is documented in neurons, whereas local translation at this site is proposed but not yet demonstrated. Inset: cryo-EM structure of the FERRY core (PDB 7ND2; PPP1R21 in blue, with CRYZL1 and GATD1); TBCK and C12orf4 are not resolved. Created in BioRender. Comisi, A. (2026) https://BioRender.com/i8p3vzq

**Fig. 2:**
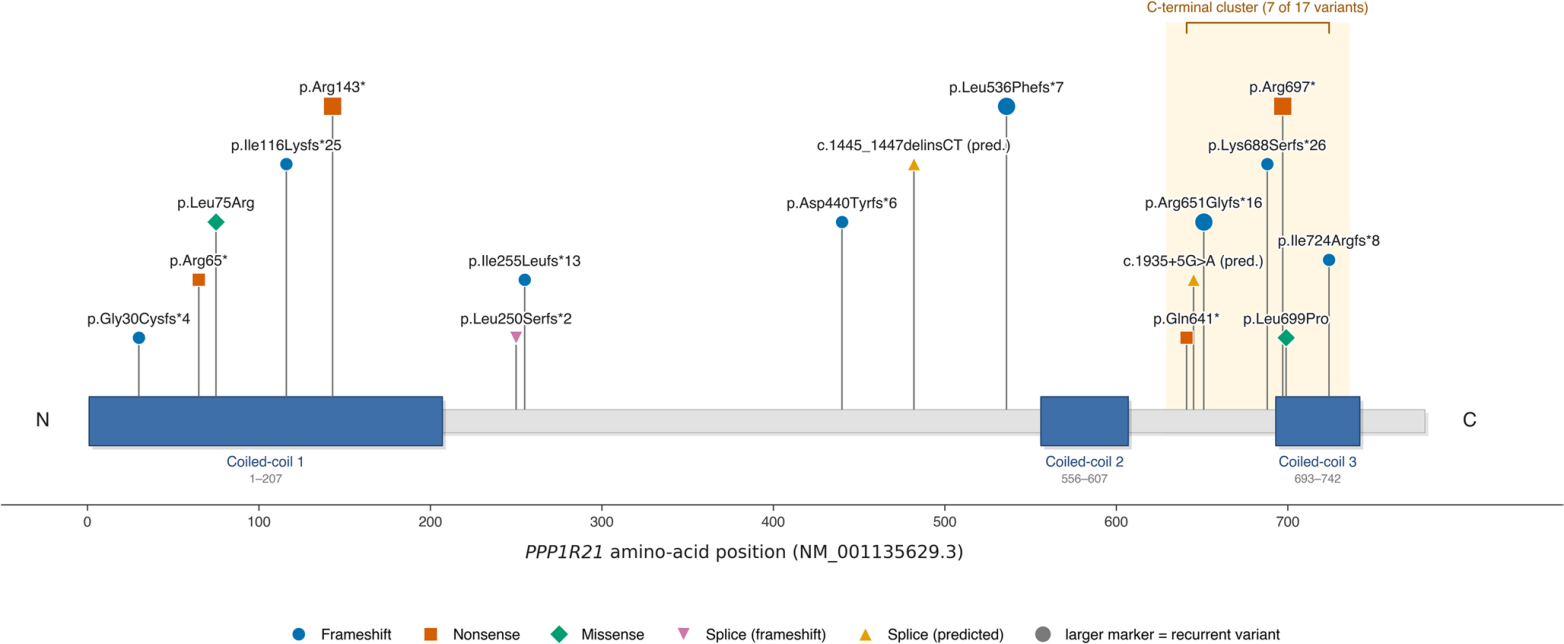
**Lollipop plot of PPP1R21 variants**. Lollipop plot of the 17 distinct PPP1R21 variants (25 individuals, 21 families) on the MANE Select transcript NM_001135629.3. The gray bar is the protein backbone; blue rectangles mark the three coiled-coil domains (aa 1–207, 556–607, 693–742), and the cream band marks the C-terminal cluster (aa 641–724). Marker shape encodes variant class (frameshift, nonsense, missense, or splice) and marker size the number of families per allele. HGVS nomenclature was normalized with VariantValidator and Mutalyzer 3; predicted splice variants are shown at approximate positions with a “(pred.)” suffix

*PPP1R21* exerts its primary cellular activity as a constituent of the FERRY complex on Rab5-positive early endosomes [4]. Functional evidence from patient-derived fibroblasts shows delayed transferrin-488 clearance with preserved uptake and near-complete co-localization with early endosome antigen 1 (EEA1) (without changes in EEA1 staining), consistent with impaired post-endocytic processing rather than internalization defects [1, 4]. Biochemical and structural studies propose that FERRY binds a subset of mRNAs via an interface largely formed by PPP1R21 coiled-coils and transports transcripts toward distal compartments to enable local translation in polarized cells such as neurons. In primary rat hippocampal neurons, PPP1R21 (within the FERRY complex) localizes to Rab5-positive early endosomes throughout the soma, dendrites, and axons, where it co-localizes (above random chance) with both mRNA, including transcripts encoding mitochondrial proteins (e.g., *MDH2*), and mitochondria; whether these endosome-associated transcripts are translated locally remains to be demonstrated [14]. In this assembly, PPP1R21 serves as a backbone within a 1:2:1:2:4 stoichiometry (TBCK: PPP1R21:C12orf4:CRYZL1:GATD1), while binding Rab5-GTP through its C-terminal coiled-coil situates the complex on early endosomes [4, 14, 15]. Notably, this same C-terminal coiled-coil also docks the two peripheral subunits TBCK and C12orf4, which map to residues ~ 646–705 of PPP1R21 immediately adjacent to the Rab5-binding site; the recurrent disease-associated truncations clustering in this region (the C-terminal variant hotspot in Fig. 2) are therefore predicted to disrupt Rab5-mediated endosomal recruitment and the incorporation of TBCK and C12orf4 in parallel, compromising both membrane targeting and complex assembly [15]. PPP1R21 is also annotated as a protein phosphatase 1 (PP1) regulatory subunit, although specific PP1 substrates remain undefined [2]. Given the broad neuronal roles of PP1, including regulation of calcium/calmodulin-dependent protein kinase II (CaMKII) relevant to learning/memory and effects on synaptic organization and cytoskeletal dynamics, *PPP1R21* may intersect with these pathways [2, 20, 21] (Fig. 1).

## Phenotypic spectrum

### Epidemiology

After deduplication, 25 affected individuals from 21 families were assembled. The sex distribution was 11 males and 14 females (M: F = 0.79), with a mean age at last evaluation of ~ 6.0 years (range ~ 0.02–27 years). Vital status was documented for 24/25; among these, 20/24 were alive and 4/24 were deceased at 7 weeks, 1.0, 1.83, and 9.5 years, underscoring premature death in a subset of cases [1–3, 5–10]. The disorder arises predominantly in consanguineous families and is uniformly homozygous at the variant level within this series: after deduplication, 21 families were identified, 20/21 reported parental consanguinity (most often first/second cousins), and all affected individuals were found to carry homozygous *PPP1R21* variants [1–3, 5–10].

Among the 24 individuals from consanguineous unions, first-cousin unions accounted for 9/24, second-cousin unions 4/24, and double first-cousin unions 1/24; in 10/24 the exact degree was not reported (“same tribe,” “cousins,” or unspecified). Geographically, cases clustered in regions with high consanguinity: Middle East (11 families across Syria, Oman, Iran, Saudi Arabia, Kuwait, Iraq), North Africa (3 across Egypt, Sudan), South Asia (2 across India, Pakistan), and Europe (2 across Italy, Germany); country of origin was not reported in the original publications for three early individuals. Within-region recurrence of specific alleles suggested founder effects, notably c.1950del (p.Arg651Glyfs*16) in unrelated Saudi families and c.1607dupT (p.Leu536Phefs*7) in Iranian cases, warranting targeted carrier studies [1, 7, 10]. Overall, the footprint, high consanguinity, homozygosity in all reported cases, and regional clustering support an autosomal-recessive architecture associated with possible local founder effects of pathogenic variants [1–3, 5–10].

### Neurological and Neurodevelopmental Features

Profound neurodevelopmental impairment was universal across these patients, with all evaluated individuals meeting criteria for global developmental delay (GDD)/ID and combined motor, cognitive, language, and social/interaction deficits [1, 2, 5–10]. Early milestones were markedly delayed: among those with explicit data, unsupported sitting was achieved by ~half (5/10; median 16 months, range 6–18 months), with additional cases attaining only supported sitting (19 months; 4 years) [1, 2, 10]. Ambulation, when acquired, was late and unstable; approximately two thirds walked (8/12; median 3.25 years, range 1.67–7.0 years) and one third never walked, typically with a broad-based and/or ataxic gait [1, 2, 10]. Expressive language was uniformly poor: absent or minimal speech (≤ 2 words) predominated (9/12), with only rare, markedly delayed phrase speech in adolescence [2, 6, 10]. Neurological examination showed a consistent profile dominated by hypotonia (22/25), occasionally with neonatal truncal hypertonia and peripheral hypotonia; deep tendon reflexes were reduced/absent in about two thirds (12/18), yielding a lower-motor-neuron–like examination despite central etiology [1, 5, 6, 9, 10]. Epilepsy affected a minority of cases (5/21 unprovoked; 7/21 including febrile-only); reported ages at onset cluster around early childhood (median ~ 4.0 years), with responses ranging from monotherapy control to pharmacoresistance [1, 8, 10]. Movement abnormalities were common and heterogeneous (9/25), most often ataxic/unsteady gait with occasional stereotypies or bruxism; overt developmental regression was not a consistent feature, and two siblings were noted to make slow but steady gains [1, 10].

### Dysmorphic features

A recognizable, age-progressive coarse craniofacial gestalt emerged, becoming more conspicuous with age (coarse facies 17/24, rising from 1/3 in infants to 3/3 in adolescents) [1, 2, 5, 6, 10]. The dominant elements were thick, high-arched eyebrows (15/24), a broad nasal bridge (10/24), a long philtrum (10/24), low-set ears (10/24), thick or everted lips (9/24), a low-broad columella (8/24), a short nose (8/24), and a high-arched palate (8/24). Generalized hypertrichosis was frequent where assessed (10/18). Less consistent findings, including hypertelorism, epicanthus, flat occiput, mandibular changes, single palmar creases, and macroglossia, were variably present across individuals [1, 2, 5, 6, 9, 10]. The complete frequency catalog and per-individual data are provided in Table 1 and Online Resource 3.

**Table 1:** Frequency of dysmorphic features in individuals with *PPP1R21*-related neurodevelopmental disorder

Dysmorphic feature	*n*/*N*	%	Dysmorphic feature	*n*/*N*	%
Coarse facies/features	17/24	70.8%	Deep-set eyes	2/24	8.3%
Thick/bushy/high/arched eyebrows	15/24	62.5%	Mandibular prognathism	2/24	8.3%
Broad nasal bridge	10/24	41.7%	Micrognathia/small chin	2/24	8.3%
Long/prominent philtrum	10/24	41.7%	Prominent nasal bridge	2/24	8.3%
Low-set ears	10/24	41.7%	Retrognathia	2/24	8.3%
Hypertrichosis	10/18	55.6%	Telecanthus	2/24	8.3%
Thick lips / everted lower lip	9/24	37.5%	Up-slanted palpebral fissures	2/24	8.3%
Low/broad columella	8/24	33.3%	Triangular face	2/24	8.3%
Short nose	8/24	33.3%	Almond-shaped eyes	1/24	4.2%
High-arched palate	8/24	33.3%	Depressed nasal bridge	1/24	4.2%
High forehead	7/24	29.2%	Broad face	1/24	4.2%
Epicanthus	6/24	25.0%	Down-slanted palpebral fissures	1/24	4.2%
Flat occiput	5/24	20.8%	Excessive drooling	1/24	4.2%
Hypertelorism	5/24	20.8%	Flat orbital ridges	1/24	4.2%
Single/deep palmar/plantar creases	4/24	16.7%	Long face	1/24	4.2%
Upturned nasal tip	4/24	16.7%	Macrocephaly/large head	1/24	4.2%
Macroglossia/protruded tongue	3/24	12.5%	Narrow nasal bridge	1/24	4.2%
Plagiocephaly/skull asymmetry	3/24	12.5%	Round face	1/24	4.2%
Bitemporal narrowing	2/24	8.3%	Thick scalp hair	1/24	4.2%
Blue sclerae	2/24	8.3%	Tented mouth	1/24	4.2%

Notes: Features are ordered by decreasing frequency. Hypertrichosis evaluated in 18/24 individuals; all other features assessed in 24/24 individuals where documentation was available

### Systemic manifestations

Multisystem involvement was frequent, spanning ophthalmologic, respiratory, gastrointestinal, cardiac, musculoskeletal, endocrine, renal/metabolic, auditory, and growth domains. The most consistent burdens were ophthalmologic (19/22; chiefly strabismus, nystagmus, and optic atrophy), gastrointestinal/feeding (18/24; dysphagia, reflux, and aspiration risk), respiratory (14/24; recurrent infections and apnea), and musculoskeletal (14/16; progressive scoliosis and contractures); a single strawberry hemangioma of the knee was also recorded [1]. Beyond the per-organ frequencies, three patterns merit note: cardiac findings ranged from transient neonatal anomalies that resolved spontaneously to structural disease (septal hypertrophy, left-ventricular noncompaction); hearing was preserved in every formally tested individual (0/6); and head circumference was heterogeneous, encompassing both microcephaly (2/24) and macrocephaly (1/24). Denominators reflect the number of individuals explicitly assessed for each domain, and per-organ frequencies and manifestations are summarized in Table 2 [1–3, 5–10].

**Table 2:** Systemic manifestations of PPP1R21-related neurodevelopmental disorder, by organ system. Denominators reflect the number of individuals explicitly assessed for each domain. Per-individual data: Online Resource 1; phenotype matrix: Online Resource 3. Abbreviations: ASD, atrial septal defect; PFO, patent foramen ovale; PDA, patent ductus arteriosus; ABR, auditory brainstem response

Organ system	*n*/*N*	%	Key manifestations
Ophthalmologic	19/22	86	Strabismus, nystagmus, optic atrophy/pallor, impaired fixation/tracking; keratitis and ptosis (one adult each)
Gastrointestinal / feeding	18/24	75	Poor suck, oropharyngeal dysphagia, drooling, aspiration risk, reflux, constipation; nasogastric/gastrostomy feeding in severe cases; hepatosplenomegaly in a subset
Respiratory	14/24	58	Neonatal distress, recurrent lower-respiratory infections, apnea; ventilatory support/tracheostomy in severe cases
Musculoskeletal	14/16	88	Progressive scoliosis, pectus deformities, contractures, delayed bone age, osteopenia with fragility fractures, joint hyperextensibility
Cardiac	6/16	38	ASD/PFO, PDA, pulmonary stenosis, septal hypertrophy/hypertrophic cardiomyopathy, left-ventricular noncompaction (adult); some neonatal findings resolved
Endocrine	3/5	60	Hypothyroidism (overt or subclinical)
Renal / metabolic	1/7	14	Cerebral salt wasting with nephrocalcinosis; persistent neutropenia (one individual)
Auditory	0/6	0	Normal ABR/clinical assessment where tested
Growth	3/12 short stature	–	Microcephaly 2/24, macrocephaly 1/24

### Natural history

The following clinical course has the caveat to be derived from cross-sectional case reports and not from prospective longitudinal studies; survivorship bias and heterogeneous follow-up duration thus limit interpretation. Importantly, early life of *PPP1R21*-mutated individuals is dominated by profound neurodevelopmental delay with substantial feeding and respiratory comorbidity; a subset achieves modest functional gains, while skeletal complications accumulate with age. At last assessment, 14/25 were 0–5 years, 8/25 were 6–12 years, and 3/25 were ≥ 13 years; mortality 4/24 occurred at 7 weeks, 1.0, 1.83, and 9.5 years, indicating life-threatening presentations in both infancy and later childhood [1, 10]. In 0–5 years, feeding impairment (poor suck, oropharyngeal dysphagia, drooling, aspiration) was common; gastrostomy was reported in 2 individuals. Respiratory morbidity ranged from recurrent infections to neonatal distress/failure, with Pediatric Intensive Care Unit admissions and tracheostomy in severe cases (one later on home bilevel positive airway pressure), underscoring an early respiratory burden [1, 3, 5, 6, 8, 10]. In 6–12 years, neurological progress was limited but present (unstable ambulation/basic self-care in some; persistent non-ambulation/minimal speech in others), with ongoing needs (drooling management, enteral feeding, treatment of breakthrough respiratory infections) and consolidation of musculoskeletal issues (scoliosis, pectus deformities, delayed bone age, joint laxity/contractures) [1, 9, 10]. By ≥ 13 years, the few cases suggest persistent functional disability with cumulative orthopedic complications and occasional cardiac involvement rather than major cognitive gains. Available cases illustrate wide-based gait at 18 years and, at 27 years, severe scoliosis, disproportionate short stature, left-ventricular noncompaction, and ocular surface disease [2, 10]. Overt developmental regression was not a consistent feature; two siblings had no regression but rather slow incremental gains [10].

### Spectrum of pathogenic variants

Variants are reported relative to the MANE Select transcript NM_001135629.3 (encoding NP_001129101.1). In the assembled cohort of 25 affected individuals from 21 families, 17 distinct *PPP1R21* variants were recorded, with a marked predominance of *loss-of-function* alleles. Nonsense, frameshift, or canonical splice changes constituted most of the catalog and a comparable share of affected individuals, aligning with an autosomal-recessive model driven by biallelic null/near-null genotypes. All patients were homozygous, and parental consanguinity was frequent, consistent with the literature [1–3, 5–10]. Recurrent alleles reinforced this pattern, including c.2089 C > T (p.Arg697*), c.427 C > T (p.Arg143*), c.1607dupT (p.Leu536Phefs*7), c.1950del (p.Arg651Glyfs*16), and c.1935 + 5G > A. The occurrence of c.1607dupT among unrelated Iranian families and c.1950del among unrelated Saudi families suggest possible founder effects [1, 7, 10]. Missense variants were uncommon, documented in three individuals and mapping to conserved segments (e.g., p.Leu75Arg, p.Leu699Pro), consistent with a hypomorphic effect that was suggested in recent studies [10]. Among the 17 distinct variants, 13 are deposited in ClinVar and classified pathogenic (11) or likely pathogenic (2), with no variants of uncertain significance or conflicting interpretations (accessed June 2026); the remaining four, not yet in public databases, were reported as pathogenic or likely pathogenic by the original authors on the basis of loss-of-function and segregation evidence (full variant list with HGVS and ClinVar/LOVD identifiers in Online Resource 1). Classifications followed American College of Medical Genetics and Genomics/Association for Molecular Pathology (ACMG/AMP) recommendations [29]; for the c.748–3 A > G splice variant, RNA sequencing confirmed a *loss-of-function* mechanism [9] (Fig. 2).

### Genotype–phenotype correlations

Sample size does not allow a detailed genotype–phenotype correlation; thus, the following observations are descriptive and warrant future clinical investigations once patients will be identified. Many affected individuals with truncating or splice-disrupting alleles ultimately achieved at least assisted ambulation, often with broad-based/ataxic gait, a profile illustrated in series from Oman/Europe and a Sudanese sibship carrying a homozygous splice variant [1, 2, 10]. By contrast, two Iraqi siblings with p.Leu699Pro (missense) never achieved autonomous walking, whereas a Pakistani individual with p.Leu75Arg walked at four years with mild ataxia, indicating that biallelic missense disease can equal or exceed the severe motor impairment observed in many LoF genotypes [10]. Expressive language outcomes were uniformly poor across categories, absent or minimal speech predominated, with only occasional limited phrase speech in adolescence [2, 10]. Neuroimaging did not segregate by allelic class: ventricular enlargement with undulating contours, reduced supratentorial white matter, corpus callosum thinning/foreshortening, and vermian hypoplasia occurred across frameshift, nonsense, and splice genotypes, suggesting a shared downstream neuroradiologic signature rather than variant-specific patterns [1, 2, 5–7, 10]. Mortality occurred in both truncating and missense disease (including early deaths in frameshift/nonsense genotypes and a later childhood death in the individual carrying the p.Leu699Pro missense variant), while epilepsy ranged from monotherapy-responsive seizures (frameshift) to pharmacoresistance (missense), indicating that clinical severity was not strictly confined to a single variant category [1, 8, 10]. Quantitatively, descriptive cross-tabulation of the 25 individuals against variant class and protein domain (Online Resources 1 and 3) reveals consistent but hypothesis-generating trends; counts are by individual, feature denominators vary because not every individual was assessed for each trait, and the two distinct missense variants (p.Leu75Arg, p.Leu699Pro) were carried by three individuals. By mechanism, mortality was broadly distributed (frameshift 2/12, nonsense 1/6, missense 1/3) and did not segregate by allelic class. Seizures were over-represented in missense disease (2/3, both with p.Leu699Pro) and absent in all six nonsense cases, with frameshift intermediate (3/8), whereas generalized hypertrichosis showed the opposite distribution (6/6 nonsense and 3/4 splice versus 1/5 frameshift and 0/3 missense), raising the possibility that residual hypomorphic protein attenuates ectodermal features. By protein domain (coiled-coil 1, aa 1–207; coiled-coil 2, aa 556–607; coiled-coil 3, aa 693–742), no variant fell within coiled-coil 2; coiled-coil 1 concentrated the early-childhood deaths (3/7); the two missense alleles fell in different domains, p.Leu699Pro in coiled-coil 3 (both carriers epileptic, one of them the single missense death) and p.Leu75Arg in coiled-coil 1 (without seizures); truncating alleles in the inter-domain linkers were uniformly in survivors (0/9 deaths); and the intronic splice variants could not be mapped to a domain. Each of these subgroup signals rests on twelve or fewer individuals, is vulnerable to ascertainment and publication bias, and requires validation in larger international cohorts. Evidence supports a core disturbance of endosomal trafficking possibly converging on a similar clinical outcome, with allelic class modulating expressivity without distinct phenotypic subgroups.

## Neuroimaging features

### Supratentorial and white-matter features

Across studies, involvement centers on corpus callosum and supratentorial white matter with accompanying ventricular changes. Callosal abnormalities were frequent (12/20), thinning (9/20), foreshortening (4/20), and occasional hypogenesis/dysmorphia, with posterior body/splenium often implicated [1, 2, 5–7, 10]. Global white-matter volume reduction (6/20) commonly co-occurred with prominent extra-axial spaces (10/20), favoring supratentorial parenchymal loss rather than obstructive hydrocephalus [1, 5–7, 10]. Ventricular enlargement was common (12/20), with a more distinctive “wavy/irregular” lateral ventricular contour noted in a subset (4/20), alongside cavum septum pellucidum (6/20) and occasional ventricular asymmetry (2/20) [1, 2, 10]. Signal-level changes included periventricular T2 hyperintensity across ages (5/20), a nonspecific finding potentially reflecting delayed myelination or gliosis, whereas explicit delayed myelination was rarely stated (1/20) and advanced diffusion metrics were unavailable. A single case labeled periventricular leukomalacia at 1 year likely represented perinatal injury rather than primary hypomyelination [1, 8–10]. The recurrent neuroimaging features and their frequencies across the 20 individuals with available MRI are summarized in Table 3.

### Posterior fossa and brainstem

Cerebellar involvement occurred in 8/20, most often vermian hypoplasia (7/20), with occasional hemispheric atrophy or mega cisterna magna (each 1/20), and retrocerebellar arachnoid cysts in two additional cases; brainstem anomalies were uncommon (2/20; small pons/brainstem dysmorphia) [2, 5, 6, 10].

**Table 3:** Neuroimaging features in PPP1R21-related neurodevelopmental disorder. Frequencies are based on the 20 of 25 individuals with reported brain MRI, with per-feature counts compiled from the primary reports. Basal-ganglia lesions were notably absent. Abbreviations: MRI, magnetic resonance imaging; T2, T2-weighted

Region	Feature	*n*/20	%
Corpus callosum	Any callosal abnormality	12/20	60
Corpus callosum	Thinning	9/20	45
Corpus callosum	Foreshortening	4/20	20
Supratentorial white matter	Volume reduction	6/20	30
Extra-axial spaces	Prominence	10/20	50
Ventricles	Enlargement	12/20	60
Ventricles	“Wavy/irregular” lateral contour	4/20	20
Septum pellucidum	Cavum	6/20	30
White-matter signal	Periventricular T2 hyperintensity	5/20	25
White-matter signal	Explicit delayed myelination	1/20	5
Cerebellum	Any involvement	8/20	40
Cerebellum	Vermian hypoplasia	7/20	35
Posterior fossa	Mega cisterna magna / arachnoid cyst	3/20	15
Brainstem	Small pons / dysmorphia	2/20	10
Basal ganglia	Lesions	0/20	0

### Pattern recognition for diagnosis

A recurrent constellation emerged: callosal thinning/foreshortening, white-matter signal/volume abnormalities often with extra-axial prominence, ventricular enlargement (frequently with cavum septum pellucidum) and vermian hypoplasia. Periventricular T2 changes were frequent but nonspecific, overt hypomyelination was rarely documented, and the basal ganglia were consistently spared. No single feature is pathognomonic; their diagnostic value lies in co-occurrence, with basal-ganglia sparing arguing against mitochondrial disease. Accordingly, in a young child with profound global developmental delay, axial hypotonia, a coarse facial gestalt, and a consanguineous background, this pattern should prompt prioritization of *PPP1R21* in exome or genome analysis [1–3, 5, 6, 8–10]. The principal differential diagnoses sharing white-matter involvement and developmental delay are TBCK-related encephaloneuropathy, EPG5-related Vici syndrome, PI4K2A-related encephalopathy, and the hypomyelinating leukodystrophies; basal-ganglia sparing and the absence of overt hypomyelination help distinguish PPP1R21-NDD from the last group [4, 22, 24]. Standardized MRI reporting of these recurrent features, particularly callosal metrics and the characteristic “wavy” lateral-ventricular contour, would improve cross-study comparability.

## Pathophysiology and disease models

### Endo-lysosomal dysfunction and the FERRY complex

*PPP1R21* deficiency primarily disrupts endo-lysosomal trafficking [1, 9, 10, 22]. In patient-derived fibroblasts, transferrin-488 internalization is normal but clearance after a 2-h chase is delayed, indicating a post-endocytic processing defect rather than impaired uptake [1]. Ultrastructurally, an increased burden of myelin-like bodies yields a lysosomal-storage–like appearance [1, 4, 9, 23]. Mechanistically, PPP1R21 is the Rab5-binding subunit and structural backbone of the FERRY complex, situating the defect within Rab5-dependent early endosomal maturation [4]. Loss of *PPP1R21* likely stalls early endosomes in an intermediate state, hindering cargo recycling and degradation [1, 4, 9, 10, 22, 24]. Mutations in *TBCK*, *PPP1R21*, and *C12orf4* have been framed as a shared nosologic group linked by the FERRY complex. In *TBCK*, autophagy–lysosome defects feature accumulation of microtubule-associated protein 1 A/1B-light chain 3 (LC3)-positive autolysosomes, indicating impaired autophagosome clearance [4]. Comparative interpretation across the three disorders remains limited by uneven case numbers and few cross-member functional assays. Emerging structural and cellular data further raise the possibility that individual FERRY components have additional, non-overlapping roles beyond the complex, a hypothesis warranting direct testing [4]. Importantly, all functional studies on *PPP1R21*-deficient cells have been performed in patient-derived fibroblasts; although FERRY-mRNA co-localization has been shown in rodent neurons [14], no *PPP1R21*-mutant neuronal model exists to date.

### Ubiquitin–proteasome system and proteostasis

Proteomics shows ubiquitin-proteasome system (UPS) activation with increased expression of multiple proteasome subunits and assembly factors (e.g., PSMB9, PSMB10, PSMA5, PSMD7, PSME2, PSMG2, PSMG4) and approximately fivefold higher chymotrypsin-like activity that resists MG132 inhibition, despite accumulation of ubiquitinated proteins; these data derive from a single patient’s fibroblasts [9]. These findings support compensatory UPS upregulation secondary to impaired autophagy–lysosome flux [25–27], generating proteostasis stress (“proteostasis crisis”).

### Cytoskeletal and structural alterations

Converging proteomic/functional data link UPS hyperactivation and proteostasis imbalance to broader structural consequences, implicating cytoskeletal organization under stress conditions [9, 25, 27].

### Mechanistic target of rapamycin (mTOR) signaling

Human *PPP1R21* reports emphasize endosomal trafficking and proteostasis rather than direct mTOR complex 1 (mTORC1)/2 readouts. In a *ppp1r21* zebrafish model of primary biliary cholangitis, rapamycin reduced hepatic immune infiltration/fibrosis and improved bile-duct pathology, suggesting context-specific crosstalk [10, 28]. By comparison, *TBCK* shows reduced mTORC1 in some systems and unchanged signaling in others, implying cell-type dependence [4]. A cross-disorder “differential mTOR” model remains unproven for *PPP1R21* and *C12orf4* and requires targeted assays in relevant human cellular models [4, 10, 28].

### Integrated translational framework for *PPP1R21*-related neurodevelopmental disorder

The preceding subsections separate robust findings in patient-derived human cells from inferences extrapolated from related models. These observations can be read as a single causal chain in which the evidence weakens from its proximal to its distal end. The proximal links are the best supported: biallelic loss or hypomorphic alleles remove the FERRY backbone and its Rab5-binding subunit, and patient fibroblasts show impaired maturation of Rab5-positive early endosomes, with delayed transferrin-488 clearance despite preserved uptake and EEA1 co-localization. The same cells accumulate ubiquitinated proteins and mount a compensatory ubiquitin–proteasome response, up-regulating PSMB9, PSMB10, PSMA5 and PSMD7 with an approximately fivefold rise in chymotrypsin-like activity, although this last finding rests on a single patient. From this endosomal defect, together with the ultrastructural myelin-like bodies, impaired cargo recycling and lysosomal delivery can be reasonably inferred. Beyond this point the chain rests on analogy rather than direct evidence: reduced FERRY-mediated mRNA delivery in neurons is supported only by rodent data, insufficient autophagy–lysosome flux is documented for *TBCK* but not yet for *PPP1R21*, and any mTORC1/2 involvement is established only in the zebrafish biliary phenotype. The terminal links stay speculative while no human neuronal model exists: a frank proteostasis crisis, cytoskeletal reorganization, and the neuronal dysfunction presumed to drive the developmental delay and the imaging changes.

## Diagnosis and clinical management

### Diagnostic algorithm

Diagnosis follows a recognizable sequence. *PPP1R21*-NDD should be suspected in any infant or child with profound global developmental delay or intellectual disability, axial hypotonia, and a coarse facial gestalt, particularly with parental consanguinity or an affected sibling. Brain MRI with T1-weighted, T2-weighted, and fluid-attenuated inversion recovery (FLAIR) sequences and a midsagittal view is then obtained to look for the recurrent constellation described above (thin or foreshortened corpus callosum; supratentorial white-matter loss with extra-axial prominence; ventriculomegaly, occasionally with a “wavy” contour and a cavum septum pellucidum; vermian hypoplasia; basal ganglia spared), while common metabolic and chromosomal mimics are excluded with a metabolic screen and chromosomal microarray. Trio whole-exome sequencing is the first-line genetic test, with whole-genome sequencing plus copy-number and runs-of-homozygosity analysis as an alternative that reaches high yields in consanguineous neurodevelopmental cohorts [7]. Biallelic *PPP1R21* variants are then confirmed on the MANE Select transcript NM_001135629.3, annotated per HGVS, and checked for parental segregation by Sanger sequencing. Interpretation follows ACMG/AMP: truncating and canonical-splice alleles usually reach pathogenic, whereas novel missense alleles require convergent rarity, conservation, in-silico and segregation evidence, with RNA studies for non-canonical splice candidates (precedent: c.748–3 A > G) [9, 29]. Finally, novel variants should be submitted to ClinVar or LOVD, and the child referred for the multidisciplinary surveillance outlined below [1–3, 5–10].

### Multidisciplinary surveillance

Morbidity and mortality concentrate in a few domains, so surveillance should be trigger-based rather than scheduled. Respiratory and feeding complications are the leading drivers of early morbidity (14/24 and 18/24) and accounted for the four deaths (4/24), warranting pulmonology with a swallow assessment and nutrition or gastroenterology input whenever apnea, recurrent infections, aspiration, or faltering growth appear, with a low threshold for gastrostomy. Otherwise, neurology and electroencephalography are indicated at the first seizure, orthopedics when scoliosis or contractures progress, cardiology when a murmur or exercise intolerance arises (and once in adolescence, given the reported cardiomyopathy), and ophthalmology for new strabismus or visual concern. Fixed intervals are not proposed, because the available evidence (*n* = 25, retrospective) cannot support an evidence-based schedule [1–3, 5–10].

### Management and family counseling

No disease-modifying therapy exists; care is supportive and multidisciplinary, targeting the complications above. Dermatologic follow-up may be considered, given a hemangioma in this cohort and a similar finding reported for a related phosphatase [1–10, 30]. Genetic counseling should address the 25% autosomal-recessive recurrence risk, with cascade carrier testing for at-risk relatives (especially consanguineous pedigrees) and prenatal or preimplantation testing once the familial variants are known [1–3, 6, 8, 10].

## Discussion

### Experimental models and biomarkers

Patient-derived fibroblasts consistently show normal transferrin uptake but delayed transferrin-488 clearance, together with myelin-like bodies on electron microscopy, findings that indicate an endo-lysosomal trafficking defect [1, 9, 31]. In vivo, a *ppp1r21* zebrafish line is lethal by ~ 15 days post-fertilization and exhibits disruption of the bile ducts, gallbladder, and liver [4, 28]. These observations nominate a pragmatic cellular biomarker, the transferrin-488 uptake/clearance assay in patient fibroblasts, for functional readouts in preclinical work [1, 31].

### Candidate therapeutic targets and translational readiness

mTOR modulation emerges from zebrafish: mTORC1/2 outputs (pS6, pAKT) are increased and rapamycin rescues the bile-duct phenotype [4, 28]. Proteostasis/UPS is a second avenue, given pronounced ubiquitin–proteasome activation in fibroblasts, although the net effect of proteasome manipulation in *PPP1R21* deficiency remains uncertain and requires careful evaluation [9, 27]. A third target is endosomal maturation, aligned with *PPP1R21*’s role in endosomal sorting/maturation and the reproducible transferrin-clearance defect [1, 10, 31].

### Research perspectives

Translating the descriptive natural history into prospective evidence will require dedicated clinical infrastructure, above all an international *PPP1R21* registry with harmonized core data elements (motor, communication, respiratory, feeding, and orthopedic milestones), interoperable and privacy-preserving data-sharing compatible with *TBCK* and *C12orf4* registries for joint analyses within the FERRY-complex disease class, standardized longitudinal MRI re-imaging, and recruitment beyond high-consanguinity settings to reduce ascertainment bias [2, 4, 7, 10]. In parallel, the fibroblast findings should be extended to disease-relevant cells, in particular patient induced pluripotent stem cell (iPSC)-derived neurons and brain organoids, complemented by transcriptomic, proteomic and metabolomic profiling, while structural studies clarify how disease variants alter FERRY-complex integrity and mRNA-cargo engagement and how PPP1R21 engages PP1 [2, 4, 5, 9, 32]. Larger and more broadly recruited cohorts are also needed to define the still poorly characterized adult phenotype, refine genotype–phenotype correlations, estimate the frequency of extra-neurologic features such as hypertrichosis and short stature, and assess cardiac, endocrine and immune involvement and the role of genetic modifiers [2, 9, 10].

### Caveats and limitations

Several constraints qualify the conclusions of this review. The total cohort (*n* = 25) precludes formal statistical inference and prevents fully defined genotype–phenotype correlations, as the identification of future cases will be crucial to dissect the *PPP1R21*-related neurodevelopmental disorder. Publication and ascertainment bias likely enrich the literature for severe phenotypes, while the predominance of consanguineous Middle Eastern and North African families limits generalizability to outbred populations. Retrospective case reporting introduces heterogeneous feature documentation, particularly for systemic and ophthalmologic findings. Adult data remain sparse, and survivorship bias (4/24 deaths during follow-up) truncates the natural-history picture. All mechanistic evidence derives from patient fibroblasts; no neuronal model yet exists, restricting extrapolation to disease-relevant cell types.

## Conclusions

*PPP1R21*-related neurodevelopmental disorder is a coherent autosomal-recessive encephalopathy driven by biallelic *loss-of-function* variants and clustered in consanguineous families. It converges on profound global developmental delay or ID with near-universal hypotonia, markedly limited expressive language, and a recognizable coarse craniofacial gestalt. Neuroimaging shows a recurring but non-pathognomonic pattern: callosal thinning or foreshortening, supratentorial white-matter loss with extra-axial prominence, ventriculomegaly, and a variably reduced vermis, with sparing of the basal ganglia. This constellation can focus genetic testing when interpreted in the appropriate clinical context. Patient fibroblasts indicate an endosomal dysfunction with secondary ubiquitin–proteasome activation; the causal hierarchy among these processes and their neuronal correlates remain unproven. Missense variants are rare and not uniformly milder, so allelic class alone does not predict outcome. Exome- or genome-based diagnosis with segregation analysis should be prioritized in suggestive consanguineous presentations, while management remains supportive and multidisciplinary. Field priorities are multicenter natural-history studies with a harmonized imaging lexicon, patient-derived neural models to test whether the fibroblast defects are recapitulated in disease-relevant cells, functional pipelines to adjudicate novel missense variants, and pathway-based interventions targeting endosomal trafficking or proteostasis, monitored by transferrin-clearance readouts.

## Supplementary Information

Below is the link to the electronic supplementary material.

Supplementary Material 1

Supplementary Material 2

Supplementary Material 3

## Data Availability

This narrative review is based entirely on previously published data. No new patient data or genetic variants were generated. PPP1R21 variant data are publicly available through ClinVar (https://www.ncbi.nlm.nih.gov/clinvar/?term=PPP1R21[gene]) and the Leiden Open Variation Database (https://databases.lovd.nl/shared/genes/PPP1R21). Individual-level clinical data extracted from published sources are provided in Online Resources 1–3.

## Abbreviations

ACMG: American College of Medical Genetics and Genomics
AMP: Association for Molecular Pathology
CaMKII: calcium/calmodulin-dependent protein kinase II
EEA1: early endosome antigen 1
FERRY: Five-subunit Endosomal Rab5 and RNA/ribosome intermediarY
FLAIR: fluid-attenuated inversion recovery
GDD: global developmental delay
GRCh38: Genome Reference Consortium Human Build 38
HGVS: Human Genome Variation Society
ID: intellectual disability
iPSC: induced pluripotent stem cell
LoF: loss of function
MANE: Matched Annotation from NCBI and EMBL-EBI
MRI: magnetic resonance imaging
mTOR: mechanistic target of rapamycin
mTORC1: mechanistic target of rapamycin complex 1
NDD: neurodevelopmental disorder
OMIM: Online Mendelian Inheritance in Man
PP1: protein phosphatase 1
PPP1R21: Protein Phosphatase 1 Regulatory subunit 21
SANRA: Scale for the Assessment of Narrative Review Articles
UPS: ubiquitin-proteasome system

## Declarations

### What is already known about this topic

Biallelic PPP1R21 variants cause a severe autosomal-recessive neurodevelopmental disorder (OMIM#619383) linked to the FERRY endosomal complex. Published case reports and small series describe global developmental delay, epilepsy, coarse facial features, and white-matter abnormalities, but no systematic phenotypic synthesis exists. Functional studies in patient fibroblasts suggest disrupted endo-lysosomal trafficking and altered proteasome activity

### What this study adds

First comprehensive review of all 25 molecularly confirmed individuals, with phenotypic frequencies, standardized denominators, and individual-level data in Online Resources 1, 2, and 3. Description of a recurrent neuroimaging pattern combining supratentorial white-matter volume loss, thin corpus callosum, and cerebellar atrophy. A proposed pathophysiological model linking endosomal mRNA transport, proteostasis, and mTOR signaling, with a structured research agenda for preclinical validation
